# Near-zero cohesion and loose packing of Bennu’s near subsurface revealed by spacecraft contact

**DOI:** 10.1126/sciadv.abm6229

**Published:** 2022-07-07

**Authors:** Kevin J. Walsh, Ronald-Louis Ballouz, Erica R. Jawin, Chrysa Avdellidou, Olivier S. Barnouin, Carina A. Bennett, Edward B. Bierhaus, Brent J. Bos, Saverio Cambioni, Harold C. Connolly, Marco Delbo, Daniella N. DellaGiustina, Joseph DeMartini, Joshua P. Emery, Dathon R. Golish, Patrick C. Haas, Carl W. Hergenrother, Huikang Ma, Patrick Michel, Michael C. Nolan, Ryan Olds, Benjamin Rozitis, Derek C. Richardson, Bashar Rizk, Andrew J. Ryan, Paul Sánchez, Daniel J. Scheeres, Stephen R. Schwartz, Sanford H. Selznick, Yun Zhang, Dante S. Lauretta

**Affiliations:** 1 Southwest Research Institute, Boulder, CO, USA.; 2 Lunar and Planetary Laboratory, University of Arizona, Tucson, AZ, USA.; 3 Johns Hopkins University Applied Physics Laboratory, Laurel, MD, USA.; 4 National Air and Space Museum, Smithsonian Institution, Washington, DC, USA.; 5 Université Côte d’Azur, Observatoire de la Côte d’Azur, CNRS, Laboratoire Lagrange, Nice, France.; 6 Lockheed Martin Space, Littleton, CO, USA.; 7 NASA Goddard Space Flight Center, Greenbelt, MD, USA.; 8 Department of Earth, Atmospheric and Planetary Sciences, Massachusetts Institute of Technology, Cambridge, MA, USA.; 9 Department of Geology, Rowan University, Glassboro, NJ, USA.; 10 Department of Astronomy, University of Maryland, College Park, MD, USA.; 11 Department of Astronomy and Planetary Science, Northern Arizona University, Flagstaff, AZ, USA.; 12 School of Physical Sciences, The Open University, Milton Keynes, UK.; 13 Colorado Center for Astrodynamics Research, University of Colorado Boulder, Boulder, CO, USA.; 14 Smead Aerospace Engineering Sciences Department, University of Colorado Boulder, Boulder, CO, USA.; 15 Planetary Science Institute, Tucson, AZ, USA.; 16 Ascending Node Technologies, Tucson, AZ, USA.

## Abstract

When the OSIRIS-REx spacecraft pressed its sample collection mechanism into the surface of Bennu, it provided a direct test of the poorly understood near-subsurface physical properties of rubble-pile asteroids, which consist of rock fragments at rest in microgravity. Here, we find that the forces measured by the spacecraft are best modeled as a granular bed with near-zero cohesion that is half as dense as the bulk asteroid. The low gravity of a small rubble-pile asteroid such as Bennu effectively weakens its near subsurface by not compressing the upper layers, thereby minimizing the influence of interparticle cohesion on surface geology. The underdensity and weak near subsurface should be global properties of Bennu and not localized to the contact point.

## Teaser

Spacecraft forces measured during touchdown indicate a weakly bound surface with twice the void space of the bulk asteroid.

## INTRODUCTION

The goal of NASA’s OSIRIS-REx (Origins, Spectral Interpretation, Resource Identification, Security–Regolith Explorer) mission was to collect and return at least 60 g of surface material from Bennu (*1*). Bennu is a small (~500 m in diameter), near-Earth asteroid with a carbonaceous composition (*2*, *3*). It has a rubble-pile structure (*4*–*6*), meaning it is composed of boulders and gravel that reaccumulated after the catastrophic disruption of a larger “parent” body (*7*).

To collect a sample in the microgravity environment of Bennu, the OSIRIS-REx spacecraft was equipped with the Touch-and-Go Sample Acquisition Mechanism (TAGSAM), an annular aluminum container (or head) with a base plate of 32 cm in diameter at the end of an extendable robotic arm (*8*). TAGSAM was designed to contact the surface for several seconds, during which it would release nitrogen gas and capture the subsequently mobilized particles in its sample collection chamber. This operation took place successfully on 20 October 2020 near the center of the ~20-m-diameter Hokioi crater in the Northern Hemisphere of Bennu (*1*). TAGSAM contacted the surface of Bennu at 10 cm s^−1^ with negligible lateral velocity. The high-pressure gas was released after 1 s of contact. After about 6 s of contact, the spacecraft initiated a back away maneuver to leave Bennu.

The low-speed contact of TAGSAM with Bennu provides a valuable probe of small-body surface properties. Previous spacecraft contacts with small-body surfaces led, in some cases, to bouncing; these interactions provided estimates for compressive strength of a cometary surface (*9*, *10*) and the coefficient of restitution of near-Earth asteroid Itokawa (*11*, *12*). In the case of TAGSAM at Bennu, the 10 cm s^−1^ contact and penetration directly probes the strength of the near-subsurface material that has otherwise been inferred from artificially created impact craters (*13*) or full-body shape and spin properties (*4*, *14*).

Measurements of craters in boulder faces (*15*) and of thermal properties (*16*–*18*) have enabled estimation of the strength and porosity of discrete particles on rubble-pile asteroids. However, the ensemble of particles (or regolith) at an asteroid’s surface—those controlling interactions with spacecraft and influencing the long-term surficial evolution—have not been probed directly. To understand the physical properties of Bennu’s near subsurface (defined here as 0 to 10 cm in depth), here, we use image and accelerometer data collected by the OSIRIS-REx spacecraft to reconstruct the interaction between TAGSAM and the regolith during the window of time where the measured forces are exclusively from the asteroid-spacecraft contact—that is, before they became dominated by the firing of the high-pressure gas into the surface.

When the spacecraft arrived at Bennu, the asteroid’s bulk density was measured to be 1190 kg m^−3^ (*4*, *19*). Studies of the boulder population have estimated a wide range of boulder porosity, up to ~55% (*17*, *20*), and lower macroporosity throughout the asteroid (as low as 12 to 15%), indicating smaller voids between larger porous constituent pieces (*21*, *22*). The bulk shape of Bennu, including some long-running ridges (*4*), suggests the existence of some cohesion or strength to provide structural shape, but with relatively low values of cohesion less than ~1 Pa (*4*, *23*). These values are consistent with expectations derived from numerical modeling for loosely packed cohesive grains of 100 μm and larger (*24*, *25*) but is less than some experimental measures with fine grains (*26*, *27*) and some inferences of 3 to 300 Pa for other rubble-pile asteroids (*14*, *24*, *28*). Bennu, unlike other small asteroids such as Eros and Itokawa (*29*), has no extensive ponded deposits of fine particles (*5*), and spectral and thermal studies have indicated minimal coverage by dust at the surface (*17*, *30*).

## RESULTS

Before, during, and after the sampling event, the Sample Acquisition Verification Camera (SamCam) of the OSIRIS-REx Camera Suite (OCAMS) (*31*) captured images looking downward over TAGSAM every 1.2 s (table S1), with a pixel scale of ~1 mm during sampling. A SamCam image preceding contact by ~30 s at 21:49:12.362 UT captured the entirety of the contacted region and shows a ~20-cm boulder extending partly into the region of contact (fig. S1). This boulder is evident in digital terrain models (DTMs) derived from combining laser altimeter data (*32*) with the stereophotoclinometry techniques (*33*), and its height above the rest of the site is about 5 cm (fig. S1).

**Fig. 1.**
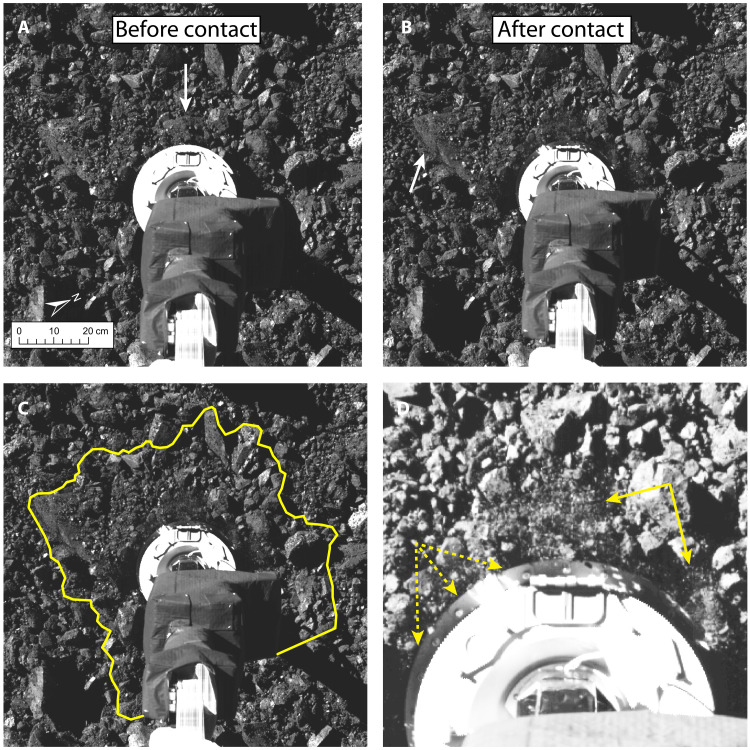
Surface changes around the circumference of TAGSAM. (**A**) Close-up view of the sample collection site just before contact at 21:49:48.882 UT. TAGSAM (round) can be seen at the end of its mechanical arm, and the arrow indicates the 20-cm rock that was first contacted. (**B**) Post-contact image at 21:49:50.101 UT with arrow indicating the lofted debris. (**C** and **D**) Same image as in (B), with a yellow boundary around the 0.51-m^2^ area that envelopes surface disturbance (C) and a zoom-in with contrast adjusted to show the lofted debris (solid arrows) and shadows over the lip of the sampler head (dashed arrows) (D). Animated version is in fig. S3 and movie S1.

The SamCam images that bracket the moment of contact were taken at 21:49:48.882 and 21:49:50.101 UT (hereafter, images SAM48 and SAM50, respectively) and show considerable disturbance at the sample site caused by contact (Fig. 1, fig. S3, and movie S1). In the latter image, nearly every visible particle is moved or reoriented at all points along the circumference of TAGSAM and stretching out to ~40 cm radially, equaling a total area of disturbance of 0.51 m^2^ compared to the TAGSAM footprint of just 0.08 m^2^.

These SamCam images also show a rock with a length of ~40 cm that has been levered upward (Fig. 1, A and B), responding to the downward force of TAGSAM. Although strong enough to withstand breaking, the rock was reoriented, and the small debris was lofted off its surface. Similarly, macroscopic debris at or below the resolution limit of the image (~1 mm per pixel) was lofted very near TAGSAM (Fig. 1D). The mobility of these millimeter-scale particles under relatively weak forces suggests minimal cohesive bonding with the surface of the larger rock.

**Fig. 2.**
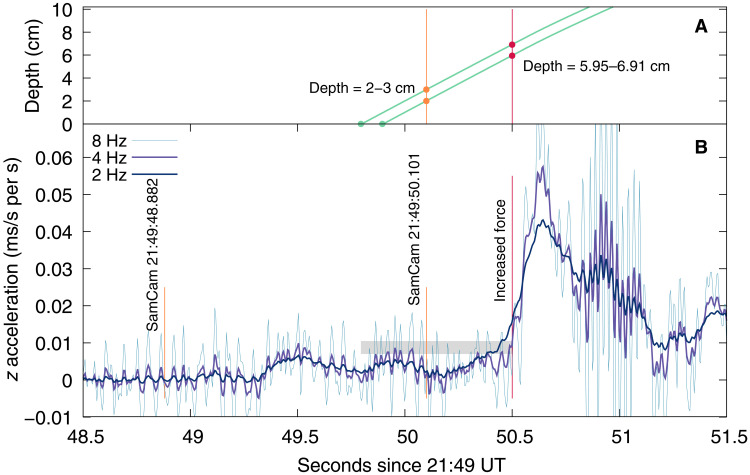
The measured accelerations during contact and the derived depth profile. (**A**) The derived depth of TAGSAM penetration into the surface, calculated by integrating the raw *z* acceleration measurements. The two profiles shown reach a depth of 2 and 3 cm, indicating approximate uncertainty bounds, at the time of the SamCam image at 21:49:50.101 UT (orange dots and vertical line). Our analysis only considers the behavior up to 21:49:50.5 UT (red dots and vertical line). (**B**) Accelerations into the surface (*z* direction) measured by the spacecraft IMU at 200 Hz and analyzed with 2-, 4-, and 8-Hz low-band frequency cutoffs (see Materials and Methods). The gray box indicates a range of force from 10 to 15 N, orange lines denote SamCam images taken before and after contact, and the red vertical line indicates where increased force due to the release of high-pressure gas begins to alter the interaction and ends the extent of the analysis presented here. A longer time series of these data is available in fig. S5.

The spacecraft’s inertial measurement unit (IMU) did not record the initial spike in acceleration that is typically observed in laboratory impact experiments into granular materials (*34*); instead, the data show a gradual increase in acceleration (Fig. 2). We attribute the gradual increase to the off-center contact with the 5-cm-tall rock (fig. S1), which resulted in a measurable change of orientation of TAGSAM and prevented the maximum force being exerted at the instant of contact. Such a change involves not only the inertia of TAGSAM itself but also the stiffness associated with cables and tubing that bridge the universal joint connecting it to the spacecraft. Fitting an ellipse to the visible outline of the TAGSAM head’s outer circumference indicates that it changed orientation by ~7° (see Materials and Methods), which would be required to accommodate the contact with the ~5-cm-tall rock. The low measured peak forces (Fig. 2) and the tilting of the head around the rock indicate that the rock was not entirely disrupted or crushed, although some damage is possible, given the inferred porosity of rocks on Bennu (*17*) and indications of lofted debris from the point of contact (Fig. 1D).

Despite of the tilt caused by contacting the rock, the TAGSAM head was in contact with the surface at all visible points along its circumference by the time SAM50 was taken, as indicated by the roughly equal disturbance of material in all directions (Fig. 1, B and C, and movie S1) and the shadows cast onto it (Fig. 1D). The top of the TAGSAM head, which is ~7 cm tall, is not shadowed by any surrounding material, but the tops of the features on the narrow lip of TAGSAM, which is 2 to 3 cm tall, are shadowed. Furthermore, measured particle sizes around the circumference of TAGSAM range from 2 to 4 cm, and given that complete shadowing would result from penetrating deeper than this characteristic size, we estimate a depth of penetration no greater than ~3 cm. Thus, by the time this image was taken, the TAGSAM head had tilted, was flush to the surface, and had penetrated 2 to 3 cm deep.

We used SAM50 as an anchor to determine the timing of contact. We integrated the measured spacecraft accelerations (Fig. 2) at different possible times of initiation of penetration to determine the contact time that achieves 2 to 3 cm of penetration at the time of SAM50. This finds that TAGSAM was flush with the surface and began penetration between 21:49:49.795 and 21:49:49.895 UT.

The high-pressure gas release was triggered just after SAM50 at 21:49:50.421 UT, and the measured pressure in the gas bottles first indicated a decrease in pressure at 21:49:50.66 UT (see Materials and Methods). Given the complexity of determining the exact timing at which the gas release affected the dynamics of the system, we use the first notable increase in measured acceleration, which occurred at 21:49:50.5 UT (Fig. 2, fig. S5, and table S1), to define the end of the pregas interaction. By the end of this interaction, TAGSAM had reached a depth of 5.95 to 6.91 cm in 0.605 to 0.705 s (see Materials and Methods). The measured accelerations never exceeded 0.014 m s^−2^, or forces above 10 to 15 N, during this time (Fig. 2). Spacecraft telemetry shows that the telescoping spring on the forearm of TAGSAM experienced no compression (see Materials and Methods). Thus, with a rigid system between the TAGSAM and the spacecraft, the acceleration profile recorded by the IMU is indicative of the forces imparted on TAGSAM by the asteroid.

The measured accelerations can be used to infer near-subsurface properties via granular physics force laws that were empirically developed to describe the forces experienced by impactors penetrating granular material at low speeds (*34*–*36*). These formulations relate the forces felt by the impactor or its final penetration depth to the geotechnical properties. In the case of TAGSAM at Bennu, the impact event involved an irregularly shaped sampling device touching down on a surface with unknown geotechnical properties, including packing fraction, (*P*), near-subsurface bulk density (ρ), cohesion (σ_c_), and angle of friction (φ).

To explore the sensitivity of the TAGSAM-regolith interaction to the properties listed above, we developed a model of the impact of a TAGSAM-shaped projectile on a regolith bed based on the *N*-body collisional codes PKDGRAV and GDC-I (*37*, *38*).The regolith is modeled as spherical particles that interact with the spacecraft and each other through a soft-sphere discrete element method (SSDEM). SSDEM allows the interacting objects to slightly overlap, enabling the modeling of multicontact frictional and cohesive forces. The friction terms include static, rolling, and twisting friction as well as a shape factor (*39*). These friction terms allow the spherical particles to mimic the behavior of rough angular particles so that the bulk φ of the system can be systematically varied. These simulations have previously been used to explore the variability in the outcome of a TAGSAM-regolith interaction for different surface geotechnical properties and impact speeds, *U* (*37*). This modeling found two distinct regimes of TAGSAM-regolith behavior for weakly cohesive regolith (σ_c_ ≤ ~10 Pa): (i) For packing fraction *P* ≤ ~0.5, the forces do not exceed ~60 N, and the force is dominated by a drag force term that depends on φ and ρ; and (ii) for *P* ≥ ~0.6, the TAGSAM forces exceed ~60 N for φ ≥ ~20°, and TAGSAM only penetrates the first few centimeters for φ ≥ ~30°.

**Fig. 3.**
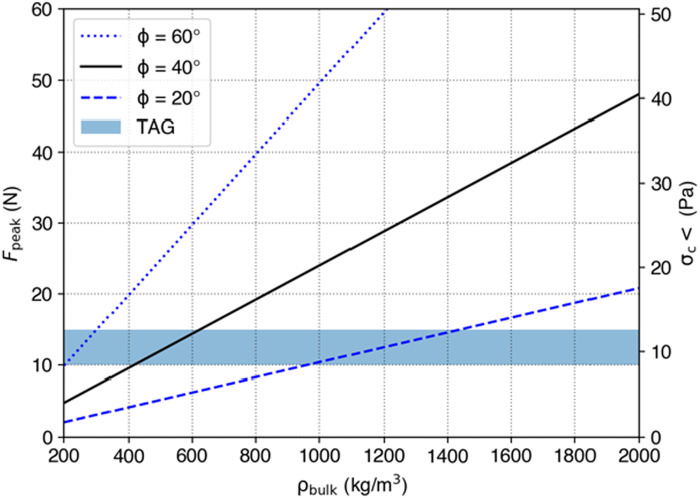
Simulations of TAGSAM penetrating granular material best fit with a low bulk density surface. A series of outcomes from numerical experiments (see Materials and Methods) produced a dependence of peak force on bulk density of the material (ρ), *F* = 0.62 tan(φ)ρ*U*^4/3^ (*37*), with the blue shaded region showing the range of measured force during contact. The relationship depends on the impact speed, cross-sectional area of the impactor (the TAGSAM head), and the determination of the angle of friction (φ) from the surrounding geology (*1*, *4*), together with the known area of TAGSAM and the velocity and force of the sampling event that constrain the bulk density of the material affected.

Given that the measured forces during the OSIRIS-REx sampling event never exceeded 10 to 15 N, our modeling indicates that the TAGSAM penetration dynamics fall in the first regime, where *P* ≲ 0.5 and, as estimated from global studies, the bulk cohesion of the near subsurface at the sampling site is less than 10 Pa (Fig. 3). In this regime, the force *F* felt by the spacecraft can be modeled by *F* = 0.62 tan(φ)ρ*U*^4/3^ (*37*). The DTMs of the sample collection site show slopes with maximum of 40° (*1*, *4*); thus, we assume this value for the angle of friction (φ ~40°). The measured pregas maximum force of 10 to 15 N would therefore require a bulk density of ρ = 440 to 600 kg m^−3^ for the uppermost 6 to 7 cm and likely the upper 10 to 20 cm that would have been immediately affected during the interaction (*37*). These findings of bulk density are consistent with independent results derived from the size of the crater formed during contact and gas release (*1*).

## DISCUSSION

These values represent underdensities of 37 to 50% relative to the bulk density of Bennu (1190 kg m^−3^). Bennu’s boulders can have a large range in microporosity as inferred from variations in thermal inertia (*17*, *20*): from about 24 to 55%, respectively, corresponding to densities of 1332 to 2249 kg m^−3^. By adopting this range of values for the particles at the sample collection site, we find that the derived ρ implies a packing fraction of 0.2 to 0.45 for the near subsurface, consistent with our assessment that *P* ≤ ~0.5. The low packing fraction is approximately half as dense as that estimated for the bulk asteroid and for the typical random packing of particles in environments where gravitational forces overwhelm cohesive bonding (*40*).

The force at contact, change in speed of the spacecraft, and the cross section of the TAGSAM head suggest compressional strength of 2 to 200 Pa in the near subsurface (see Materials and Methods), similar to the value derived for the surface of comet 67P/Churyumov-Gerasimenko (*10*). This compressive strength corresponds to a bulk cohesion of approximately 0.2 to 20 Pa (*41*), which are orders of magnitude less than the cohesion measured on larger airless bodies, which can be as high as 4000 Pa, e.g., at the Moon (*42*). Nevertheless, they are supported by other lines of evidence. The size of the excavated region from the gas release at TAG requires a nearly cohesionless material (<1 Pa) (*1*), and the presence of an extensive ejecta blanket around Bralgah crater on Bennu implies that the effective strength of surface material is ≤2 Pa (*43*). On the rubble-pile asteroid Ryugu, the Hayabusa2 mission’s artificial impact experiment (*13*) indicated a similarly weak surface with an effective strength of <1.3 Pa. Stability analyses of Bennu’s surface have suggested that the terraces (*44*) and other geomorphologic indicators of mass movement observed globally (*45*) would be suppressed if cohesion were above ~0.6 Pa (*44*).

Why does Bennu have so little cohesion in its near subsurface? The cohesive bonding force depends on particle sizes, roughness, and filling factors. Larger numbers of smaller particles and flatter rock surfaces serve to increase the total contact surface area and bonding strength (*46*, *47*). Image analysis of macroscopic shape properties of boulders (*48*) reveals no substantial difference between the axial ratios of Bennu boulders compared to other airless bodies in the solar system [e.g., Ryugu boulders (*49*) and lunar boulders (*50*)]. Future microscopic analysis of the returned samples may shed more light on the effect of particle roughness on the near-surface geotechnical properties. Neither mid-infrared emission spectra (*30*) nor diurnal temperature curves (*17*) indicated any substantial presence of surface dust on Bennu; the former dataset is sensitive to dust coatings on the surface, and the latter constrains the percentage of dust to less than 10% of material down to a few thermal skin depths (a few centimeters). However, a dust cloud was observed immediately after gas was released during sampling (*1*). The cloud contained an estimated 1.8 kg of material from a 60-cm-diameter area, with an equivalent layer thickness of 3.2 (−2.2/+3.2) mm (*1*). The TAGSAM head had penetrated at least 6 to 7 cm before gas release; so if the liberated dust resided throughout the volume of ~0.02 m^3^, then it would account for ~20% of the total mass of material, exceeding the constraints from thermal analysis (*17*). Alternatively, the observed cloud could contain material liberated from much deeper and/or the dust preferentially resided below the thermal skin depth and remained undetected by the thermal observations. Both are consistent with the finding of the low packing fraction and low strength at the near subsurface.

The very low packing fraction derived here for the near subsurface would serve to decrease particle contacts and provide pathways for particle percolation into the subsurface. If a large reservoir of fine particles exists in the subsurface—as suggested by the dust cloud shown in (*1*)—then it could contribute to stronger bonding and global strength at depth. Some strength at depth has been suggested on the basis of relatively shallow depth/diameter ratios of craters >80 m wide and the presence of mounds at the centers of some craters (*51*); in addition, the artificial impact experiment on Ryugu hinted at higher strength with depth (*13*). Furthermore, observations of the crater created by sampling, which excavated the subsurface to a depth of 68 ± 10 cm, show an increase in visible particles smaller than 90 cm, relative to the presampling surface; this suggests that the subsurface contains more small particles than the surface, which could similarly increase strength and packing fraction with depth (*1*). Nevertheless, the spectral and thermal properties indicating a scarcity of dust in the uppermost centimeters of Bennu’s surface are not localized to the Hokioi crater from which the sample was collected, which supports the notion of a global lack of near-subsurface strength.

A loosely packed near subsurface is in general agreement with previous laboratory work on particle settling in reduced gravity (*52*) and with the possibility of low-density regions near the surface from gravity studies at Bennu (*53*). The paucity of particle contacts implied by low-filling-factor packing means that the surface and near subsurface will be more easily reorganize than the more densely packed interior of the asteroid, resulting in the surface being effectively weaker. A low filling factor has important consequences for the surface evolution of an asteroid. Models of asteroid evolution under the dynamic changes of spin-up forced by the Yarkovsky-O’Keefe-Radzievskii-Paddack effect (*7*) find that surfaces that are weaker or less dense than the interiors result in surficial slope failure instead of internal failure deep within the asteroid (*39*, *53*, *54*). Surface destabilization is observed in Bennu’s midlatitudes as evidenced by material moving toward its equator (*44*, *45*) and at lower latitudes on Ryugu moving away from its equator (*55*), suggesting that the low near-subsurface filling factor might be a global property of these rubble-pile asteroids and not localized to the singular spot on Bennu contacted by the TAGSAM head. Furthermore, the contacted spot in Hokioi crater was selected specifically for its abundance of collectable fine (<2-cm) particles (*1*, *48*), so it is possible that the rest of Bennu’s surface has less of this material and therefore even less strength. However, this spot is also within a relatively young crater with a high slope, posing potentially unique geologic considerations.

Previous theory has proposed that the average regolith particle size increases as asteroid size decreases owing to the more efficient retention of impact-comminuted material by bodies of larger size and thus greater surface gravity (*56*). However, this simple correlation between surface particle size and gravity is belied by the dichotomy between the carbonaceous rubble piles Bennu and Ryugu and the stony rubble pile Itokawa; all three are subkilometer in size, but the former two are boulder dominated with no extensive ponded deposits of small particles on their surfaces [e.g., (*5*, *55*)], whereas 20% of the latter’s surface is covered by ponded particles; these ponds are not impact craters but rather geopotential lows that capture material moving downslope (*11*). Thus, the ability to support and maintain a macroporous structure in the near subsurface may leave a pathway for the percolation of small particles into the interior of the asteroid over time. With even lower strength, the near subsurface may simply compress under gravity and retain fine particles in ponds on the surface. This implies that Itokawa’s regolith cannot support low packing fraction near-surface structures, and instead the near surface has compressed and frustrated the percolation of fine particles. This could also be the outcome of a competition between production and percolation, where different rates of regolith production for different materials (*20*, *57*) could compete against, or potentially dominate, a percolation effect, or that ponded deposits are simply recently revealed subsurface layers following a disruptive reorganization of the body (*46*).

## MATERIALS AND METHODS

### Image acquisition and processing

SamCam images were collected before, during, and after sampling on 20 October 2020, with a range to the surface of the asteroid that changed rapidly during the maneuvers. The two images recorded at 21:49:48.882 and 21:49:50.101 UT were captured at slightly different range from the surface. The local solar time at the time of the images was approximately 3:10 p.m., and the phase angle was approximately 60°, with an emission angle of 16° and incidence angle of 71°. The pixel scale for both images was approximately the same at ~1.5 mm per pixel. Both images shown in Fig. 1 were registered to a common shape model (v20) with mean facet size of 5 cm that was created using laser altimetry (*6*). SamCam images followed the standard OCAMS calibration detailed in (*58*) and were only used for geologic context so were not photometrically corrected.

### Spacecraft telemetry

The on-board IMUs measured the accelerations of the spacecraft in 200 Hz before, during, and immediately after contact with Bennu. The IMU hardware shares heritage with the Phoenix lander spacecraft (*59*, *60*). The raw telemetry in the *z* direction is available in data file S1 at 21:49:49 to 21:49:50.5 UT. The conversion to newton is done for a spacecraft mass of 1391 kg.

Three bottles with high-pressure nitrogen gas were attached the extendable arm between the spacecraft and TAGSAM (*8*). Bottle no. 3 initially had 60.7 g of gas at 2.0105 × 10^7^ Pa (2916 psi) before venting and was the only bottle used during the interaction. The pressure in the bottle of high-pressure Nitrogen gas shows its first decrease in pressure at 21:49:50.66 UT (fig. S4). Notably, this value was recorded at 10 Hz, and thus no more granularity in the measurement is available for comparison with the acceleration data that were recorded at 200 Hz.

There is a telescoping spring located on the arm between the spacecraft and TAGSAM (*1*, *8*) [figure 16 in (*8*)]. This telescoping spring is a constant-force spring that was designed to compress at forces above ~60 N, although other cabling and blanketing was expected to increase the minimum force required to begin compression. The spring was outfitted with a microswitch to indicate compression of the spring (*1*, *8*). The spacecraft telemetry indicated that the microswitch was never triggered, and the spring was therefore not compressed during the interaction with the asteroid.

### Topography of the sample collection site

The topography of the sampling location is investigated with a DTM constructed from stereophotoclinometry techniques (*33*) combined with laser altimetry (*6*, *32*, *33*, *61*). The DTM has a resolution of approximately 2 cm and is displayed as a colored shaded relief with the lighting of the scene similar to that during the sampling event (*1*). A series of chords across the sampling site show that the large contacted boulder sits only 4 to 5 cm above the lowest parts of the sampling site (fig. S1).

### Ellipse fitting of TAGSAM

The outline of the TAGSAM head was analyzed in a sequence of images to estimate its relative change in orientation during the sampling event. The sequence of images just before and immediately after sampling was analyzed in the SAOImageDS9 computer application (*62*), where the visible outer edge of the TAGSAM head was mapped with points (*N* ≥ ~35) in a sequence of images (fig. S2). The final fits were found to be accurate within ~0.5 pixel for finding the center of the ellipse and ~1 pixel for the semi-axes lengths of the ellipse. The ratio of the semi-major and semi-minor axes was used to calculate the tilt of the circular TAGSAM head out of the image plane, finding a change of 6.8° average for two separate measurements from before contact at 21:49:46.446 UT to the image at contact at 21:49:50.101 UT (table S2). The semi-major axis changed orientation from ~26° counterclockwise from +*y* to ~90°. The large boulder that was contacted was in the +*y* direction, and thus its location and presumed contact would explain reorienting TAGSAM +*y* upward in the post-contact image so that its visible long axis was then 90° away from +*y*.

### TAGSAM tilt to accommodate a 5-cm-tall rock

TAGSAM is 32 cm in diameter (base plate), and thus a 5-cm rock would induce a tilt of ${\text{sin}}^{-1}(\frac{5\;\text{cm}}{32\;\text{cm}})=\sim 9^{{}^{\circ}}$ under perfectly static conditions. The recorded 7° tilt would require a minimum a 4-cm-tall obstruction, and thus a 5-cm obstruction is tall enough to provide the measured tilt.

### Estimation of compression strengths

A simple upper limit on the compressive strength at the location of contact is given by the measured forces divided by the cross-sectional area of the TAG head, equaling 125 to 187.5 Pa for the 10 to 15 N forces and a 0.08-m^2^ area.

An energetics approach finds that the compressional strength is bounded by the change in kinetic energy of the impactor divided by the compressed volume (*10*). With a change in speed of 0.4 cm/s over the first 0.7 s of the interaction that compressed a minimum of 0.08-m^2^ area cylinder, ~7 cm deep gives a maximum compressive strength of ~2 Pa. The estimated compressive strengths are an order of magnitude larger than the maximum estimated bulk cohesion of the material, which is in line with expectations for their relationship (*41*).

### Numerical modeling

The numerical model used in this work constructed low filling factor granular beds (corresponding to a large macroporosity and substantial void spaces between particles), rather than using a nominally packed bed of lower density material (representing a large microporosity). While the two could have identical bulk densities, the former allows for reorganization and compression of minimal near subsurface before particle contacts increase to levels equal to the interior of the asteroid. There is no evidence on Bennu for extremely low-density/highly microporous particles such as the few extraordinary boulders on Ryugu that have been detected through their thermal signature and estimated to have porosity >70% (*18*). Rather, thermal investigation found the two most common boulder types have density between 1332 and 2249 kg m^−3^, which does not imply microporosity above 55%. Studies of the particle size distributions reach similar conclusions on boulder density of ~50% but estimate that the most porous boulders are the most abundant, leading to an asteroid macroporosity of 12 to 15% (*21*, *22*).

## References

[1] D. S. Lauretta, C. D. Adam, A. J. Allen, R.-L. Ballouz, O. S. Barnouin, K. J. Becker, T. Becker, C. A. Bennett, E. B. Bierhaus, B. J. Bos, R. D. Burns, H. Campins, Y. Cho, P. R. Christensen, E. C. A. Church, B. E. Clark, H. C. Connolly Jr., M. G. Daly, D. N. DellaGiustina, C. Y. Drouet d’Aubigny, J. P. Emery, H. L. Enos, S. Freund Kasper, J. B. Garvin, K. Getzandanner, D. R. Golish, V. E. Hamilton, C. W. Hergenrother, H. H. Kaplan, L. P. Keller, E. J. Lessac-Chenen, A. J. Liounis, H. Ma, L. K. McCarthy, B. D. Miller, M. C. Moreau, T. Morota, D. S. Nelson, J. O. Nolau, R. Olds, M. Pajola, J. Y. Pelgrift, A. T. Polit, M. A. Ravine, D. C. Reuter, B. Rizk, B. Rozitis, A. J. Ryan, E. M. Sahr, N. Sakatani, J. A. Seabrook, S. H. Selznick, M. A. Skeen, A. A. Simon, S. Sugita, K. J. Walsh, M. M. Westermann, C. M. V. Wolner, K. Yumoto, Spacecraft sample collection and subsurface excavation of asteroid(101955) Bennu. Science 376, eabm1018 (2022). 10.1126/science.abm1018 35857591

[2] V. E. Hamilton, A. A. Simon, P. R. Christensen, D. C. Reuter, B. E. Clark, M. A. Barucci, N. E. Bowles, W. V. Boynton, J. R. Brucato, E. A. Cloutis, H. C. Connolly Jr., K. L. D. Hanna, J. P. Emery, H. L. Enos, S. Fornasier, C. W. Haberle, R. D. Hanna, E. S. Howell, H. H. Kaplan, L. P. Keller, C. Lantz, J.-Y. Li, L. F. Lim, T. J. M. Coy, F. Merlin, M. C. Nolan, A. Praet, B. Rozitis, S. A. Sandford, D. L. Schrader, C. A. Thomas, X.-D. Zou, D. S. Lauretta; OSIRIS-REx Team, Evidence for widespread hydrated minerals on asteroid (101955) Bennu. Nat. Astron. 3, 332–340 (2019). 31360777 10.1038/s41550-019-0722-2 PMC6662227

[3] A. A. Simon, H. H. Kaplan, V. E. Hamilton, D. S. Lauretta, H. Campins, J. P. Emery, M. A. Barucci, D. N. D. Giustina, D. C. Reuter, S. A. Sandford, D. R. Golish, L. F. Lim, A. Ryan, B. Rozitis, C. A. Bennett, Widespread carbon-bearing materials on near-Earth asteroid (101955) Bennu. Science 370, eabc3522 (2020). 33033153 10.1126/science.abc3522

[4] O. S. Barnouin, M. G. Daly, E. E. Palmer, R. W. Gaskell, J. R. Weirich, C. L. Johnson, M. M. Al Asad, J. H. Roberts, M. E. Perry, H. C. M. Susorney, R. T. Daly, E. B. Bierhaus, J. A. Seabrook, R. C. Espiritu, A. H. Nair, L. Nguyen, G. A. Neumann, C. M. Ernst, W. V. Boynton, M. C. Nolan, C. D. Adam, M. C. Moreau, B. Risk, C. D. D’Aubigny, E. R. Jawin, K. J. Walsh, P. Michel, S. R. Schwartz, R.-L. Ballouz, E. M. Mazarico, D. J. Scheeres, J. McMahon, W. Bottke, S. Sugita, N. Hirata, N. Hirata, S. Watanabe, K. N. Burke, D. N. D. Guistina, C. A. Bennett, D. S. Lauretta; OSIRIS-REx Team, Shape of (101955) Bennu indicative of a rubble pile with internal stiffness. Nat. Geosci. 12, 247–252 (2019). 31080497 10.1038/s41561-019-0330-x PMC6505705

[5] K. J. Walsh, E. R. Jawin, R.-L. Ballouz, O. S. Barnouin, E. B. Bierhaus, H. C. Connolly Jr., J. L. Molaro, T. J. M. Coy, M. Delbo’, C. M. Hartzell, M. Pajola, S. R. Schwartz, D. Trang, E. Asphaug, K. J. Becker, C. B. Beddingfield, C. A. Bennett, W. F. Bottke, K. N. Burke, B. C. Clark, M. G. Daly, D. N. D. Giustina, J. P. Dworkin, C. M. Elder, D. R. Golish, A. R. Hildebrand, R. Malhotra, J. Marshall, P. Michel, M. C. Nolan, M. E. Perry, B. Rizk, A. Ryan, S. A. Sandford, D. J. Scheeres, H. C. M. Susorney, F. Thuillet, D. S. Lauretta; OSIRIS-REx Team, Craters, boulders and regolith of (101955) Bennu indicative of an old and dynamic surface. Nat. Geosci. 12, 242–246 (2019).

[6] M. G. Daly, O. S. Barnouin, J. A. Seabrook, J. Roberts, C. Dickinson, K. J. Walsh, E. R. Jawin, E. E. Palmer, R. Gaskell, J. Weirich, T. Haltigin, D. Gaudreau, C. Brunet, G. Cunningham, P. Michel, Y. Zhang, R.-L. Ballouz, G. Neumann, M. E. Perry, L. Philpott, M. M. Al Asad, C. L. Johnson, C. D. Adam, J. M. Leonard, J. L. Geeraert, K. Getzandanner, M. C. Nolan, R. T. Daly, E. B. Bierhaus, E. Mazarico, B. Rozitis, A. J. Ryan, D. N. D. Giustina, B. Rizk, H. C. M. Susorney, H. L. Enos, D. S. Lauretta, Hemispherical differences in the shape and topography of asteroid (101955) Bennu. Sci. Adv. 6, eabd3649 (2020). 33033038 10.1126/sciadv.abd3649 PMC7544500

[7] K. J. Walsh, Rubble pile asteroids. Annu. Rev. Aston. Astrophys. 56, 593–624 (2018).

[8] E. B. Bierhaus, B. C. Clark, J. W. Harris, K. S. Payne, R. D. Dubisher, D. W. Wurts, R. A. Hund, R. M. Kuhns, T. M. Linn, J. L. Wood, A. J. May, J. P. Dworkin, E. Beshore, D. S. Lauretta; OSIRIS-REx Team, The OSIRIS-REx spacecraft and the Touch-and-Go sample acquisition mechanism (TAGSAM). Space Sci. Rev. 214, 107 (2018).

[9] J. Biele, S. Ulamec, M. Maibaum, R. Roll, L. Witte, E. Jurado, P. Muñoz, W. Arnold, H. U. Auster, C. Casas, C. Faber, C. Fantinati, F. Finke, H. H. Fischer, K. Geurts, C. Güttler, P. Heinisch, A. Herique, S. Hviid, G. Kargl, M. Knapmeyer, J. Knollenberg, W. Kofman, N. Kömle, E. Kührt, V. Lommatsch, S. Mottola, R. Pardo de Santayana, E. Remetean, F. Scholten, K. J. Seidensticker, H. Sierks, T. Spohn, The landing(s) of Philae and inferences about comet surface mechanical properties. Science 349, eaaa9816 (2015). 10.1126/science.aaa9816 26228158

[10] L. O’Rourke, P. Heinisch, J. Blum, S. Fornasier, G. Filacchione, H. Van Hoang, M. Ciarniello, A. Raponi, B. Gundlach, R. A. Blasco, B. Grieger, K.-H. Glassmeier, M. Küppers, A. Rotundi, O. Groussin, D. Bockelée-Morvan, H.-U. Auster, N. Oklay, G. Paar, M. del Pilar Caballo Perucha, G. Kovacs, L. Jorda, J.-B. Vincent, F. Capaccioni, N. Biver, J. W. Parker, C. Tubiana, H. Sierks, The Philae lander reveals low-strength primitive ice inside cometary boulders. Nature 586, 697–701 (2020). 33116289 10.1038/s41586-020-2834-3

[11] H. Yano, T. Kubota, H. Miyamoto, T. Okada, D. Scheeres, Y. Takagi, K. Yoshida, M. Abe, S. Abe, O. Barnouin-Jha, A. Fujiwara, S. Hasegawa, T. Hashimoto, M. Ishiguro, M. Kato, J. Kawaguchi, T. Mukai, J. Saito, S. Sasaki, M. Yoshikawa, Touchdown of the Hayabusa Spacecraft at the Muses Sea on Itokawa. Science 312, 1350–1353 (2006). 16741113 10.1126/science.1126164

[12] S. Van Wal, “High-fidelity simulation of small-body lander/rover spacecraft,” thesis, University of Colorado Boulder, CO (2018).

[13] M. Arakawa, T. Saiki, K. Wada, K. Ogawa, T. Kadono, K. Shirai, H. Sawada, K. Ishibashi, R. Honda, N. Sakatani, Y. Iijima, C. Okamoto, H. Yano, Y. Takagi, M. Hayakawa, P. Michel, M. Jutzi, Y. Shimaki, S. Kimura, Y. Mimasu, T. Toda, H. Imamura, S. Nakazawa, H. Hayakawa, S. Sugita, T. Morota, S. Kameda, E. Tatsumi, Y. Cho, K. Yoshioka, Y. Yokota, M. Matsuoka, M. Yamada, T. Kouyama, C. Honda, Y. Tsuda, S. Watanabe, M. Yoshikawa, S. Tanaka, F. Terui, S. Kikuchi, T. Yamaguchi, N. Ogawa, G. Ono, K. Yoshikawa, T. Takahashi, Y. Takei, A. Fujii, H. Takeuchi, Y. Yamamoto, T. Okada, C. Hirose, S. Hosoda, O. Mori, T. Shimada, S. Soldini, R. Tsukizaki, T. Iwata, M. Ozaki, M. Abe, N. Namiki, K. Kitazato, S. Tachibana, H. Ikeda, N. Hirata, N. Hirata, R. Noguchi, A. Miura, An artificial impact on the asteroid (162173) Ryugu formed a crater in the gravity-dominated regime. Science 368, 67–71 (2020). 32193363 10.1126/science.aaz1701

[14] B. Rozitis, E. Maclennan, J. P. Emery, Cohesive forces prevent the rotational breakup of rubble-pile asteroid (29075) 1950 DA. Nature 512, 174–176 (2014). 25119234 10.1038/nature13632

[15] R.-L. Ballouz, K. J. Walsh, O. S. Barnouin, D. N. D. Giustina, M. Al Asad, E. R. Jawin, M. G. Daly, W. F. Bottke, P. Michel, C. Avdellidou, M. Delbo, R. T. Daly, E. Asphaug, C. A. Bennett, E. B. Bierhaus, H. C. Connolly Jr., D. R. Golish, J. L. Molaro, M. C. Nolan, M. Pajola, B. Rizk, S. R. Schwartz, D. Trang, C. W. V. Wolner, D. S. Lauretta, Bennu’s near-Earth lifetime of 1.75 million years inferred from craters on its boulders. Nature 587, 205–209 (2020). 33106686 10.1038/s41586-020-2846-z

[16] M. Grott, J. Knollenberg, M. Hamm, K. Ogawa, R. Jaumann, K. A. Otto, M. Delbo, P. Michel, J. Biele, W. Neumann, M. Knapmeyer, E. Kührt, H. Senshu, T. Okada, J. Helbert, A. Maturilli, N. Müller, A. Hagermann, N. Sakatani, S. Tanaka, T. Arai, S. Mottola, S. Tachibana, I. Pelivan, L. Drube, J. B. Vincent, H. Yano, C. Pilorget, K. D. Matz, N. Schmitz, A. Koncz, S. E. Schröder, F. Trauthan, M. Schlotterer, C. Krause, T. M. Ho, A. Moussi-Soffys, Low thermal conductivity boulder with high porosity identified on C-type asteroid (162173) Ryugu. Nat. Astron. 3, 971–976 (2019).

[17] B. Rozitis, A. J. Ryan, J. P. Emery, P. R. Christensen, V. E. Hamilton, A. A. Simon, D. C. Reuter, M. Al Asad, R.-L. Ballouz, J. L. Bandfield, O. S. Barnouin, C. A. Bennett, M. Bernacki, K. N. Burke, S. Cambioni, B. E. Clark, M. G. Daly, M. Delbo, D. N. D. Giustina, C. M. Elder, R. D. Hanna, C. W. Haberle, E. S. Howell, D. R. Golish, E. R. Jawin, H. H. Kaplan, L. F. Lim, J. L. Molaro, D. P. Munoz, M. C. Nolan, B. Rizk, M. A. Siegler, H. C. M. Susorney, K. J. Walsh, D. S. Lauretta, Asteroid (101955) Bennu’s weak boulders and thermally anomalous equator. Sci. Adv. 6, eabc3699 (2020). 33033037 10.1126/sciadv.abc3699 PMC7544501

[18] N. Sakatani, S. Tanaka, T. Okada, T. Fukuhara, L. Riu, S. Sugita, R. Honda, T. Morota, S. Kameda, Y. Yokota, E. Tatsumi, K. Yumoto, N. Hirata, A. Miura, T. Kouyama, H. Senshu, Y. Shimaki, T. Arai, J. Takita, H. Demura, T. Sekiguchi, T. G. Müller, A. Hagermann, J. Biele, M. Grott, M. Hamm, M. Delbo, W. Neumann, M. Taguchi, Y. Ogawa, T. Matsunaga, T. Wada, S. Hasegawa, J. Helbert, N. Hirata, R. Noguchi, M. Yamada, H. Suzuki, C. Honda, K. Ogawa, M. Hayakawa, K. Yoshioka, M. Matsuoka, Y. Cho, H. Sawada, K. Kitazato, T. Iwata, M. Abe, M. Ohtake, S. Matsuura, K. Matsumoto, H. Noda, Y. Ishihara, K. Yamamoto, A. Higuchi, N. Namiki, G. Ono, T. Saiki, H. Imamura, Y. Takagi, H. Yano, K. Shirai, C. Okamoto, S. Nakazawa, Y. Iijima, M. Arakawa, K. Wada, T. Kadono, K. Ishibashi, F. Terui, S. Kikuchi, T. Yamaguchi, N. Ogawa, Y. Mimasu, K. Yoshikawa, T. Takahashi, Y. Takei, A. Fujii, H. Takeuchi, Y. Yamamoto, C. Hirose, S. Hosoda, O. Mori, T. Shimada, S. Soldini, R. Tsukizaki, M. Ozaki, S. Tachibana, H. Ikeda, M. Ishiguro, H. Yabuta, M. Yoshikawa, S. Watanabe, Y. Tsuda, Anomalously porous boulders on (162173) Ryugu as primordial materials from its parent body. Nat. Astron. 5, 766–774 (2021).

[19] D. J. Scheeres, J. W. McMahon, A. S. French, D. N. Brack, S. R. Chesley, D. Farnocchia, Y. Takahashi, J. M. Leonard, J. Geeraert, B. Page, P. Antreasian, K. Getzandanner, D. Rowlands, E. M. Mazarico, J. Small, D. E. Highsmith, M. Moreau, J. P. Emery, B. Rozitis, M. Hirabayashi, P. Sánchez, S. Van wal, P. Tricarico, R.-L. Ballouz, C. L. Johnson, M. M. Al Asad, H. C. M. Susorney, O. S. Barnouin, M. G. Daly, J. A. Seabrook, R. W. Gaskell, E. E. Palmer, J. R. Weirich, K. J. Walsh, E. R. Jawin, E. B. Bierhaus, P. Michel, W. F. Bottke, M. C. Nolan, H. C. Connolly Jr., D. S. Lauretta; OSIRIS-REx Team, The dynamic geophysical environment of (101955) Bennu based on OSIRIS-REx measurements. Nat. Astron. 3, 352–361 (2019). 32601603 10.1038/s41550-019-0721-3 PMC7323631

[20] S. Cambioni, M. Delbo, G. Poggiali, C. Avdellidou, A. J. Ryan, J. D. P. Deshapriya, E. Asphaug, R.-L. Ballouz, M. A. Barucci, C. A. Bennett, W. F. Bottke, J. R. Brucato, K. N. Burke, E. Cloutis, D. N. DellaGiustina, J. P. Emery, B. Rozitis, K. J. Walsh, D. S. Lauretta, Fine-regolith production on asteroids controlled by rock porosity. Nature 598, 49–52 (2021). 34616055 10.1038/s41586-021-03816-5

[21] J. Biele, K. N. Burke, M. Grott, A. J. Ryan, D. DellaGiustina, B. Rozitis, P. Michel, S. Schroeder, W. Neumann, Macroporosity and grain density of rubble pile asteroid (101955) Bennu, in *Proceedings of the American Geophysical Union Fall Meeting 2020* (AGU, 2020).

[22] P. Tricarico, D. J. Scheeres, A. S. French, J. W. McMahon, D. N. Brack, J. M. Leonard, P. Antreasian, S. R. Chesley, D. Farnocchia, Y. Takahashi, E. M. Mazarico, D. Rowlands, D. Highsmith, K. Getzandanner, M. Moreau, C. L. Johnson, L. Philpott, E. B. Bierhaus, K. J. Walsh, O. S. Barnouin, E. E. Palmer, J. R. Weirich, R. W. Gaskell, M. G. Daly, J. A. Seabrook, M. C. Nolan, D. S. Lauretta, Internal rubble properties of asteroid (101955) Bennu. Icarus 370, 114665 (2021).

[23] M. Hirabayashi, R. Nakano, E. Tatsumi, K. J. Walsh, O. S. Barnouin, P. Michel, C. M. Hartzell, D. T. Britt, S. Sugita, S. I. Watanabe, W. F. Bottke, D. J. Scheeres, R. L. Ballouz, Y. Cho, T. Morota, E. S. Howell, D. S. Lauretta, Spin-driven evolution of asteroids’ top-shapes at fast and slow spins seen from (101955) Bennu and (162173) Ryugu. Icarus 352, 113946 (2020).

[24] D. J. Scheeres, C. M. Hartzell, P. Sánchez, M. Swift, Scaling forces to asteroid surfaces: The role of cohesion. Icarus 210, 968–984 (2010).

[25] P. Sánchez, D. J. Scheeres, The strength of regolith and rubble pile asteroids. Meteorit. Planet. Sci. 49, 788–811 (2014).

[26] B. Gundlach, K. P. Schmidt, C. Kreuzig, D. Bischoff, F. Rezaei, S. Kothe, J. Blum, B. Grzesik, E. Stoll, The tensile strength of ice and dust aggregates and its dependence on particle properties. Mon. Not. R. Astron. Soc. 479, 1273–1277 (2018).

[27] J. Brisset, T. Miletich, J. Metzger, A. Rascon, A. Dove, J. Colwell, Multi-particle collisions in microgravity: Coefficient of restitution and sticking threshold for systems of mm-sized particles. Astron. Astrophys. 631, A35 (2019).

[28] Y. Zhang, D. C. Richardson, O. S. Barnouin, P. Michel, S. R. Schwartz, R. L. Ballouz, Rotational failure of rubble-pile bodies: Influences of shear and cohesive strengths. Astrophys. J. 857, 15 (2018).

[29] N. Murdoch, P. Sánchez, S. R. Schwartz, H. Miyamoto, Asteroid surface geophysics, in *Asteroids IV*, P. Michel, F. DeMeo, W. F. Bottke, Eds. (University of Arizona Press, 2015), pp. 767–792.

[30] V. E. Hamilton, P. R. Christensen, H. H. Kaplan, C. W. Haberle, A. D. Rogers, T. D. Glotch, L. B. Breitenfeld, C. A. Goodrich, D. L. Schrader, T. J. McCoy, C. Lantz, R. D. Hanna, A. A. Simon, J. R. Brucato, B. E. Clark, D. S. Lauretta, Evidence for limited compositional and particle size variation on asteroid (101955) Bennu from thermal infrared spectroscopy. Astron. Astrophys. 650, A120 (2021).

[31] B. Rizk, C. Drouet d’Aubigny, D. Golish, C. Fellows, C. Merrill, P. Smith, M. S. Walker, J. E. Hendershot, J. Hancock, S. H. Bailey, D. N. DellaGiustina, D. S. Lauretta, R. Tanner, M. Williams, K. Harshman, M. Fitzgibbon, W. Verts, J. Chen, T. Connors, D. Hamara, A. Dowd, A. Lowman, M. Dubin, R. Burt, M. Whiteley, M. Watson, T. McMahon, M. Ward, D. Booher, M. Read, B. Williams, M. Hunten, E. Little, T. Saltzman, D. Alfred, S. O’Dougherty, M. Walthall, K. Kenagy, S. Peterson, B. Crowther, M. L. Perry, C. See, S. Selznick, C. Sauve, M. Beiser, W. Black, R. N. Pfisterer, A. Lancaster, S. Oliver, C. Oquest, D. Crowley, C. Morgan, C. Castle, R. Dominguez, M. Sullivan, OCAMS: The OSIRIS-REx Camera Suite. Space Sci. Rev. 214, 26 (2018).

[32] M. G. Daly, O. S. Barnouin, C. Dickinson, J. Seabrook, C. L. Johnson, G. Cunningham, T. Haltigin, D. Gaudreau, C. Brunet, I. Aslam, A. Taylor, E. B. Bierhaus, W. Boynton, M. Nolan, D. S. Lauretta, The OSIRIS-REx Laser Altimeter (OLA) investigation and instrument. Space Sci. Rev. 212, 899–924 (2017).

[33] R. W. Gaskell, O. S. Barnouin-Jha, D. J. Scheeres, A. S. Konopliv, T. Mukai, S. Abe, J. Saito, M. Ishiguro, T. Kubota, T. Hashimoto, J. Kawaguchi, M. Yoshikawa, K. Shirakawa, T. Kominato, N. Hirata, H. Demura, Characterizing and navigating small bodies with imaging data. Meteorit. Planet. Sci. 43, 1049–1061 (2008).

[34] N. Murdoch, M. Drilleau, C. Sunday, F. Thuillet, A. Wilhelm, G. Nguyen, Y. Gourinat, Low-velocity impacts into granular material: Application to small-body landing. Mon. Not. R. Astron. Soc. 503, 3460–3471 (2021).

[35] J. S. Uehara, M. A. Ambroso, R. P. Ojha, D. J. Durian, Low-speed impact craters in loose granular media. Phys. Rev. Lett. 90, 194301 (2003). 12785950 10.1103/PhysRevLett.90.194301

[36] H. Katsuragi, D. J. Durian, Drag force scaling for penetration into granular media. Phys. Rev. E 87, 052208 (2013). 10.1103/PhysRevE.87.052208 23767531

[37] R.-L. Ballouz, K. J. Walsh, P. Sánchez, K. A. Holsapple, P. Michel, D. J. Scheeres, Y. Zhang, D. C. Richardson, O. S. Barnouin, M. C. Nolan, E. B. Bierhaus, H. C. Connolly, S. R. Schwartz, O. Çelik, M. Baba, D. S. Lauretta, Modified granular impact force laws for the OSIRIS-REx touchdown on the surface of asteroid (101955) Bennu. Mon. Not. R. Astron. Soc. 507, 5087–5105 (2021).

[38] P. Sánchez, D. J. Scheeres, E. B. Bierhaus, B. Clark, Simulations of regolith interactions in microgravity, in *Proceedings of the 44th Lunar and Planetary Science Conference* (2013); https://www.lpi.usra.edu/meetings/lpsc2013/pdf/2271.pdf.

[39] Y. Zhang, D. C. Richardson, O. S. Barnouin, C. Maurel, P. Michel, S. R. Schwartz, R. L. Ballouz, L. A. M. Benner, S. P. Naidu, J. Li, Creep stability of the proposed AIDA mission target 65803 Didymos: I. Discrete cohesionless granular physics model. Icarus 294, 98–123 (2017).

[40] R. P. Zou, M. L. Gan, A. B. Yu, Prediction of the porosity of multi-component mixtures of cohesive and non-cohesive particles. Chem. Eng. Sci. 66, 4711–4721 (2011).

[41] J. Biele, S. Ulamec, L. Richter, J. Knollenberg, E. Kührt, D. Möhlmann, The putative mechanical strength of comet surface material applied to landing on a comet. Acta Astronaut. 65, 1168–1178 (2009).

[42] J. K. Mitchell, L. G. Bromwell, W. D. Carrier III, N. C. Costes, R. F. Scott, Soil mechanical properties at the Apollo 14 site. J. Geophys. Res. 77, 5641–5664 (1972).

[43] M. E. Perry, O. S. Barnouin, R. T. Daly, E. B. Bierhaus, R.-L. Ballouz, K. J. Walsh, M. G. Daly, D. N. Della Giustina, M. C. Nolan, J. P. Emery, M. M. Al Asad, C. L. Johnson, C. M. Ernst, E. R. Jawin, P. Michel, D. R. Golish, W. F. Bottke, J. A. Seabrook, D. S. Lauretta, Low surface strength of the asteroid Bennu inferred from impact ejecta deposit. Nat. Geosci. 10.1038/s41561-022-00937-y (2022).

[44] O. S. Barnouin, M. G. Daly, J. A. Seabrook, Y. Zhang, F. Thuillet, P. Michel, J. H. Roberts, R. T. Daly, M. E. Perry, H. C. M. Susorney, E. R. Jawin, R.-L. Ballouz, K. J. Walsh, M. M. Sevalia, M. M. Al Asad, C. L. Johnson, E. B. Bierhaus, R. W. Gaskell, E. E. Palmer, J. Weirich, B. Rizk, C. Y. D. D’Aubigny, M. C. Nolan, D. N. D. Giustina, D. J. Scheeres, J. W. M. Mahon, H. C. Connolly Jr., D. C. Richardson, C. W. V. Wolner, D. S. Lauretta, The formation of terraces on asteroid (101955) Bennu. JGR Planets 127, e2021JE006927 (2022).

[45] E. R. Jawin, K. J. Walsh, O. S. Barnouin, T. J. McCoy, R.-L. Ballouz, D. N. D. Giustina, H. C. Connolly Jr., J. Marshall, C. Beddingfield, M. C. Nolan, J. L. Molaro, C. A. Bennett, D. J. Scheeres, M. G. Daly, M. Al Asad, R. T. Daly, E. B. Bierhaus, H. C. M. Susorney, H. H. Kaplan, H. L. Enos, D. S. Lauretta, Global patterns of recent mass movement on asteroid (101955) Bennu. J. Geophys. Res. 125, e06475 (2020).

[46] P. Sánchez, D. J. Scheeres, Rotational evolution of self-gravitating aggregates with cores of variable strength. Planet. Space Sci. 157, 39–47 (2018).

[47] M. Kiuchi, A. M. Nakamura, Relationship between regolith particle size and porosity on small bodies. Icarus 239, 291–293 (2014).

[48] K. J. Walsh, E. B. Bierhaus, D. S. Lauretta, M. C. Nolan, R. L. Ballouz, C. A. Bennett, E. R. Jawin, O. S. Barnouin, K. Berry, K. N. Burke, B. Brodbeck, R. Burns, B. C. Clark, B. E. Clark, S. Cambioni, H. C. Connolly Jr., M. G. Daly, M. Delbo, D. N. DellaGiustina, J. P. Dworkin, H. L. Enos, J. P. Emery, P. Gay, D. R. Golish, V. E. Hamilton, R. Hoover, M. Lujan, T. McCoy, R. G. Mink, M. C. Moreau, J. Nolau, J. Padilla, M. Pajola, A. T. Polit, S. J. Robbins, A. J. Ryan, S. H. Selznick, S. Stewart, C. W. V. Wolner, Assessing the sampleability of Bennu’s surface for the OSIRIS-REx asteroid sample return mission. Space Sci. Rev. 218, 20 (2022). 35528719 10.1007/s11214-022-00887-2 PMC9018658

[49] T. Michikami, C. Honda, H. Miyamoto, M. Hirabayashi, A. Hagermann, T. Irie, K. Nomura, C. M. Ernst, M. Kawamura, K. Sugimoto, E. Tatsumi, T. Morota, N. Hirata, T. Noguchi, Y. Cho, S. Kameda, T. Kouyama, Y. Yokota, R. Noguchi, M. Hayakawa, N. Hirata, R. Honda, M. Matsuoka, N. Sakatani, H. Suzuki, M. Yamada, K. Yoshioka, H. Sawada, R. Hemmi, H. Kikuchi, K. Ogawa, S.-I. Watanabe, S. Tanaka, M. Yoshikawa, Y. Tsuda, S. Sugita, Boulder size and shape distributions on asteroid Ryugu. Icarus 331, 179–191 (2019).

[50] N. E. Demidov, A. T. Basilevsky, Height-to-diameter ratios of moon rocks from analysis of Lunokhod-1 and -2 and Apollo 11-17 panoramas and LROC NAC images. Solar Syst. Res. 48, 324–329 (2014).

[51] R. T. Daly, E. B. Bierhaus, O. S. Barnouin, M. G. Daly, J. A. Seabrook, J. H. Roberts, C. M. Ernst, M. E. Perry, H. Nair, R. C. Espiritu, E. E. Palmer, R. W. Gaskell, J. R. Weirich, H. C. M. Susorney, C. L. Johnson, K. J. Walsh, M. C. Nolan, E. R. Jawin, P. Michel, D. Trang, D. S. Lauretta, The morphometry of impact craters on Bennu. Geophys. Res. Lett. 47, e89672 (2020).

[52] R. Schräpler, J. Blum, I. von Borstel, C. Güttler, The stratification of regolith on celestial objects. Icarus 257, 33–46 (2015).

[53] D. J. Scheeres, A. S. French, P. Tricarico, S. R. Chesley, Y. Takahashi, D. Farnocchia, J. W. McMahon, D. N. Brack, A. B. Davis, R.-L. Ballouz, E. R. Jawin, B. Rozitis, J. P. Emery, A. J. Ryan, R. S. Park, B. P. Rush, N. Mastrodemos, B. M. Kennedy, J. Bellerose, D. P. Lubey, D. Velez, A. T. Vaughan, J. M. Leonard, J. Geeraert, B. Page, P. Antreasian, E. Mazarico, K. Getzandanner, D. Rowlands, M. C. Moreau, J. Small, D. E. Highsmith, S. Goossens, E. E. Palmer, J. R. Weirich, R. W. Gaskell, O. S. Barnouin, M. G. Daly, J. A. Seabrook, M. M. Al Asad, L. C. Philpott, C. L. Johnson, C. M. Hartzell, V. E. Hamilton, P. Michel, K. J. Walsh, M. C. Nolan, D. S. Lauretta, Heterogeneous mass distribution of the rubble-pile asteroid (101955) Bennu. Sci. Adv. 6, eabc3350 (2020). 33033036 10.1126/sciadv.abc3350 PMC7544499

[54] M. Hirabayashi, D. P. Sánchez, D. J. Scheeres, Internal structure of asteroids having surface shedding due to rotational instability. Astrophys. J. 808, 63 (2015).

[55] S. Sugita, R. Honda, T. Morota, S. Kameda, H. Sawada, E. Tatsumi, M. Yamada, C. Honda, Y. Yokota, T. Kouyama, N. Sakatani, K. Ogawa, H. Suzuki, T. Okada, N. Namiki, S. Tanaka, Y. Iijima, K. Yoshioka, M. Hayakawa, Y. Cho, M. Matsuoka, N. Hirata, N. Hirata, H. Miyamoto, D. Domingue, M. Hirabayashi, T. Nakamura, T. Hiroi, T. Michikami, P. Michel, R. L. Ballouz, O. S. Barnouin, C. M. Ernst, S. E. Schröder, H. Kikuchi, R. Hemmi, G. Komatsu, T. Fukuhara, M. Taguchi, T. Arai, H. Senshu, H. Demura, Y. Ogawa, Y. Shimaki, T. Sekiguchi, T. G. Müller, A. Hagermann, T. Mizuno, H. Noda, K. Matsumoto, R. Yamada, Y. Ishihara, H. Ikeda, H. Araki, K. Yamamoto, S. Abe, F. Yoshida, A. Higuchi, S. Sasaki, S. Oshigami, S. Tsuruta, K. Asari, S. Tazawa, M. Shizugami, J. Kimura, T. Otsubo, H. Yabuta, S. Hasegawa, M. Ishiguro, S. Tachibana, E. Palmer, R. Gaskell, L. le Corre, R. Jaumann, K. Otto, N. Schmitz, P. A. Abell, M. A. Barucci, M. E. Zolensky, F. Vilas, F. Thuillet, C. Sugimoto, N. Takaki, Y. Suzuki, H. Kamiyoshihara, M. Okada, K. Nagata, M. Fujimoto, M. Yoshikawa, Y. Yamamoto, K. Shirai, R. Noguchi, N. Ogawa, F. Terui, S. Kikuchi, T. Yamaguchi, Y. Oki, Y. Takao, H. Takeuchi, G. Ono, Y. Mimasu, K. Yoshikawa, T. Takahashi, Y. Takei, A. Fujii, C. Hirose, S. Nakazawa, S. Hosoda, O. Mori, T. Shimada, S. Soldini, T. Iwata, M. Abe, H. Yano, R. Tsukizaki, M. Ozaki, K. Nishiyama, T. Saiki, S. Watanabe, Y. Tsuda, The geomorphology, color, and thermal properties of Ryugu: Implications for parent-body processes. Science 364, 252–252 (2019). 30890587 10.1126/science.aaw0422 PMC7370239

[56] B. Gundlach, J. Blum, A new method to determine the grain size of planetary regolith. Icarus 223, 479–492 (2013).

[57] J. L. Molaro, K. J. Walsh, E. R. Jawin, R.-L. Ballouz, C. A. Bennett, D. N. DellaGiustina, D. R. Golish, C. Drouet d’Aubigny, B. Rizk, S. R. Schwartz, R. D. Hanna, S. J. Martel, M. Pajola, H. Campins, A. J. Ryan, W. F. Bottke, D. S. Lauretta, In situ evidence of thermally induced rock breakdown widespread on Bennu’s surface. Nat. Commun. 11, 2913 (2020). 32518333 10.1038/s41467-020-16528-7 PMC7283247

[58] D. R. Golish, C. Drouet d’Aubigny, B. Rizk, D. N. DellaGiustina, P. H. Smith, K. Becker, N. Shultz, T. Stone, M. K. Barker, E. Mazarico, E. Tatsumi, R. W. Gaskell, L. Harrison, C. Merrill, C. Fellows, B. Williams, S. O’Dougherty, M. Whiteley, J. Hancock, B. E. Clark, C. W. Hergenrother, D. S. Lauretta, Ground and in-flight calibration of the OSIRIS-REx camera suite. Space Sci. Rev. 216, 12 (2020). 32025061 10.1007/s11214-019-0626-6 PMC6979463

[59] B. L. Garavelli, L. Marradi, A. Morgan, Space qualified GPS receiver and MIMU for an autonomous on-board guidance and navigation package. Proc. SPIE 2583, 539–547 (1995).

[60] P. A. Taylor, D. C. Catling, M. Daly, C. S. Dickinson, H. P. Gunnlaugsson, A.-M. Harri, C. F. Lange, Temperature, pressure, and wind instrumentation in the Phoenix meteorological package. J. Geophys. Res. 113, E00A10 (2008).

[61] O. S. Barnouin, M. G. Daly, E. E. Palmer, C. L. Johnson, R. W. Gaskell, M. al Asad, E. B. Bierhaus, K. L. Craft, C. M. Ernst, R. C. Espiritu, H. Nair, G. A. Neumann, L. Nguyen, M. C. Nolan, E. Mazarico, M. E. Perry, L. C. Philpott, J. H. Roberts, R. J. Steele, J. Seabrook, H. C. M. Susorney, J. R. Weirich, D. S. Lauretta, Digital terrain mapping by the OSIRIS-REx mission. Planet. Space Sci. 180, 104764 (2020).

[62] W. A. Joye, E. Mandel, New Features of SAOImage DS9, in *Proceedings of the Astronomical Data Analysis Software and Systems XII ASP Conference Series* (ADASS, 2003), vol. 295, pp. 489.

[63] B. Rizk, C. Drouet d’Aubigny, D. Golish, D. N. DellaGiustina, D. S. Lauretta, Origins, Spectral Interpretation, Resource Identification, Security, Regolith Explorer (OSIRIS-REx): OSIRIS-REx Camera Suite (OCAMS) bundle (NASA Planetary Data System, 2019).

