# Supplementary material for: Near-zero cohesion and loose packing of Bennu’s near subsurface revealed by spacecraft contact
Source: Sci Adv. 2022 Jul 7;8(27):eabm6229. doi: 10.1126/sciadv.abm6229 (PMC9262326; doi:10.1126/sciadv.abm6229)
Supplement: Supplementary file 1 — Figs. S1 to S5 Tables S1 and S2 [file sciadv.abm6229_sm.pdf]

Science Advances
AAAS

Supplementary Materials for

Near-zero cohesion and loose packing of Bennu's near subsurface revealed by spacecraft contact

Kevin J. Walsh *et al.*

Corresponding author: Kevin J. Walsh, kwalsh@boulder.swri.edu

Sci. Adv. **8**, eabm6229 (2022)
DOI: 10.1126/sciadv.abm6229

The PDF file includes:

Figs. S1 to S5
Tables S1 and S2
Legend for movie S1
Legend for data S1

Other Supplementary Material for this manuscript includes the following:

Movie S1
Data S1

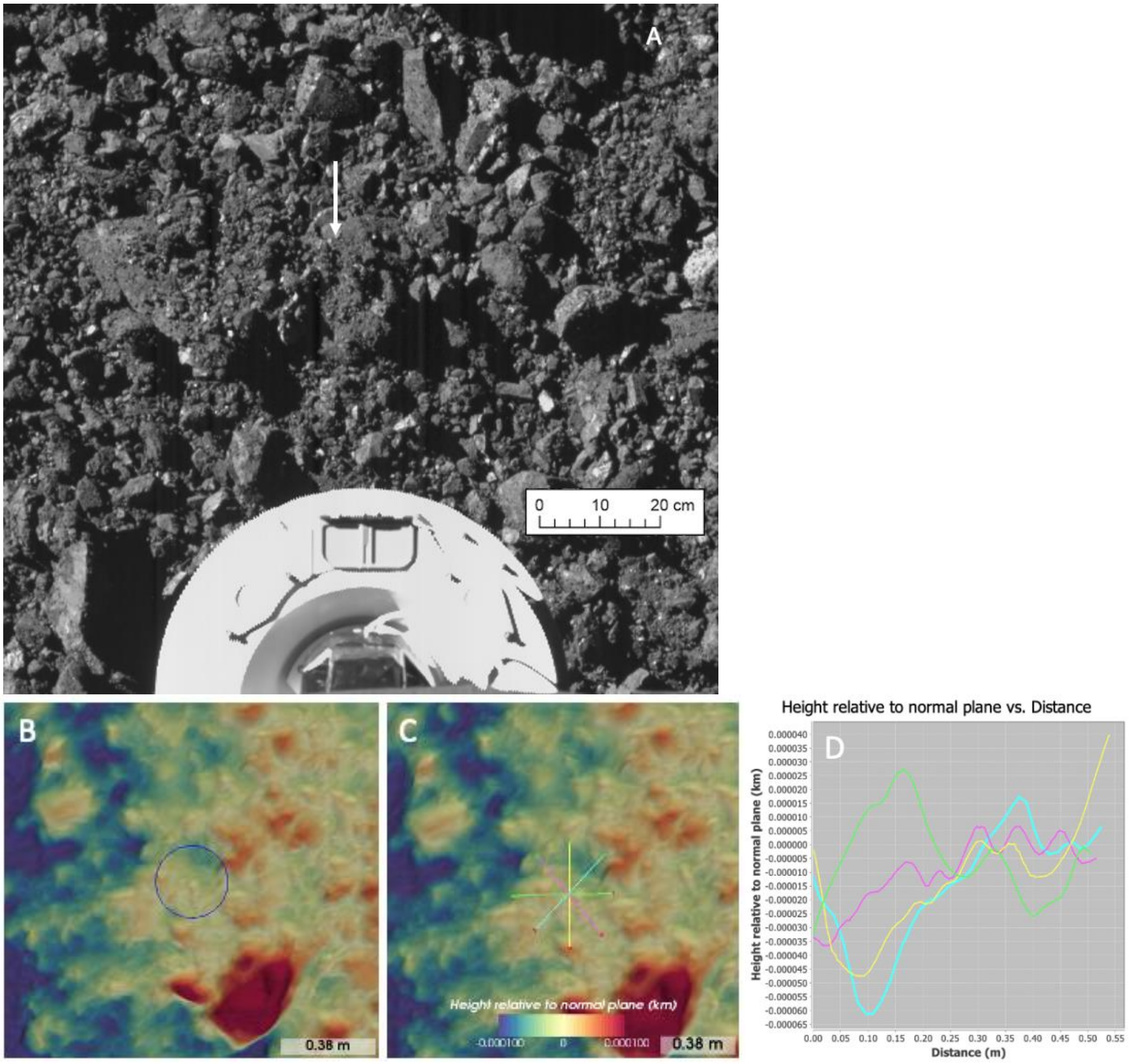

Fig. S1. Pre-contact image and the digital terrain model of the sampling site colored relative to the height above the average plane of the region. (A) Image 20201020T214917S231_sam taken at 21:49:17.231 UTC that shows the contacted spot and the 5cm tall boulder indicated by the white arrow. Note that the scale bar applies to the asteroid surface and not the TAGSAM head which is not yet in contact. (**B**) The local region of sampling with the pink circle indicating the outline of TAGSAM contact location. (**C**) Chords across the sample site and (**D**) the height profile of the chords where the green chord finds a 4–5 cm height above the lowest regions of the sample site.

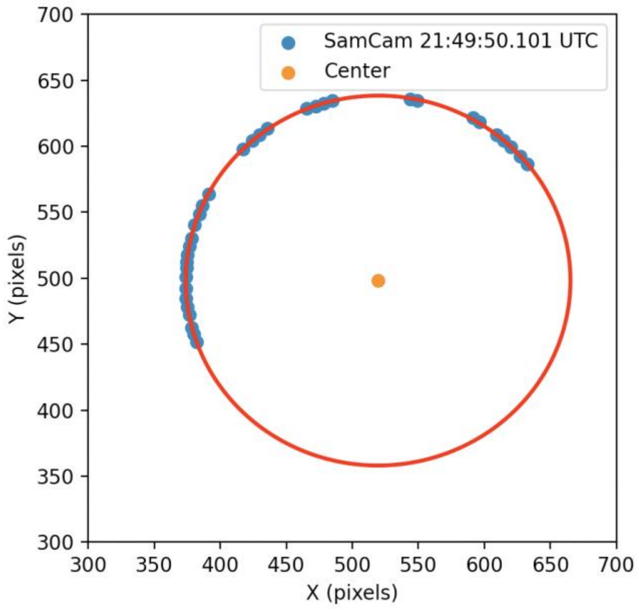

Fig. S2. The mapped points and fitted ellipse for the SamCam image taken at 21:49:50.101 UTC in units of image pixels. The mapped points show the extent around the circumference of the head where its edge was clearly visible and the fit of the ellipse through those points. The calculation for relative change in the orientation of TAGSAM relied on changes in the fitted ellipse in the units of pixel values shown here.

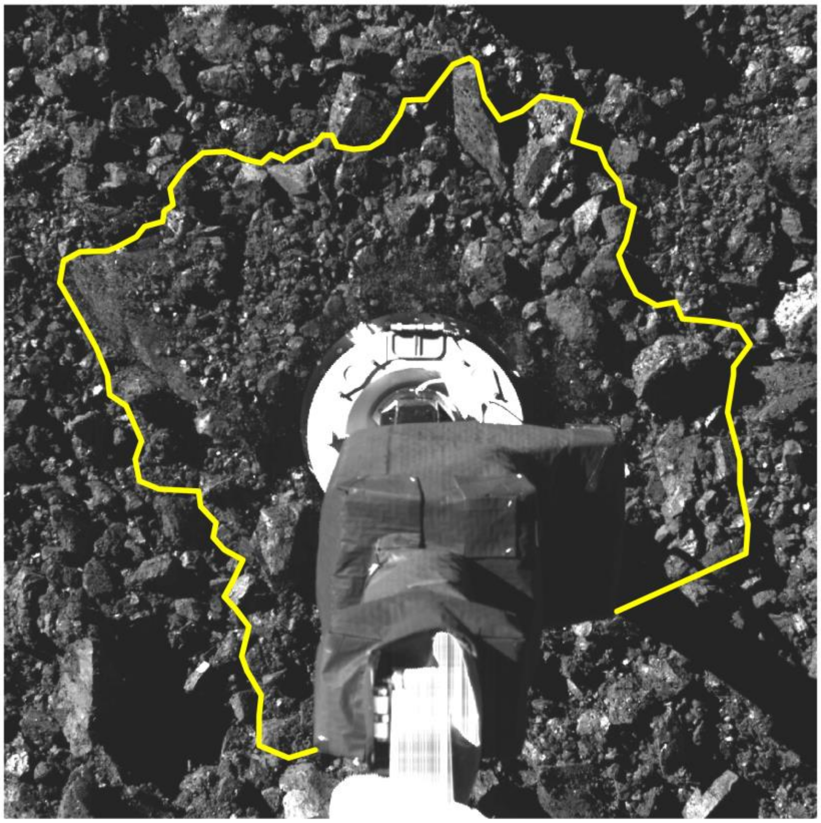

Fig. S3. Surface changes around the circumference of the TAGSAM head. Close-up view of the sample collection site just before contact at 21:49:48.882 UTC and at 21:49:50.101 UTC with a yellow boundary around the 0.51 m^2 area that envelopes surface disturbance. (Alternative to this animated gif can be found in movie S1.)

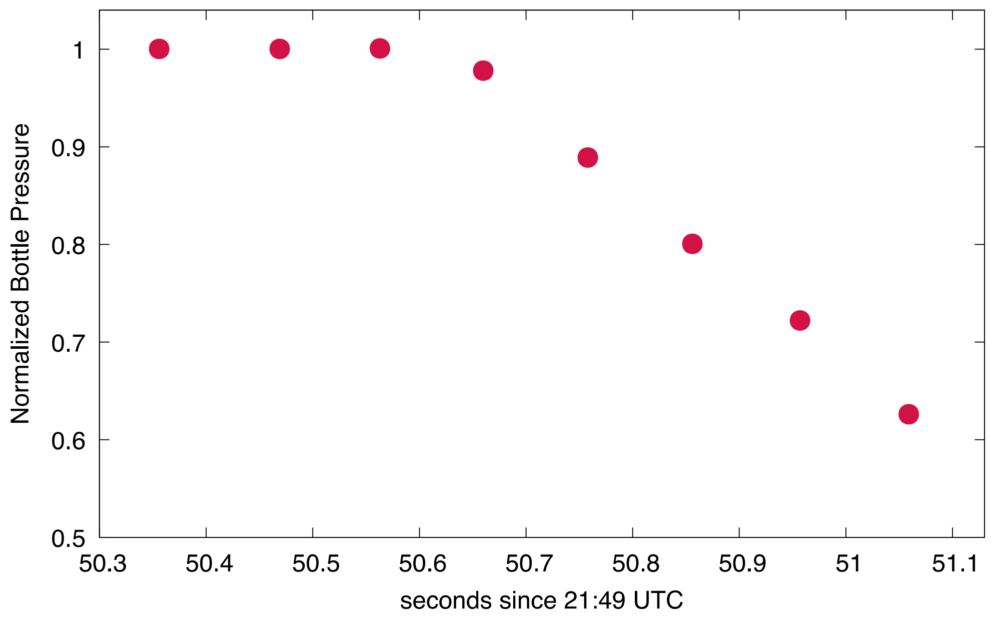

Fig S4. **Pressure in the bottle of nitrogen gas over time**. The bottle of nitrogen gas shows its first decrease in pressure at 21:49:50.66 UTC.

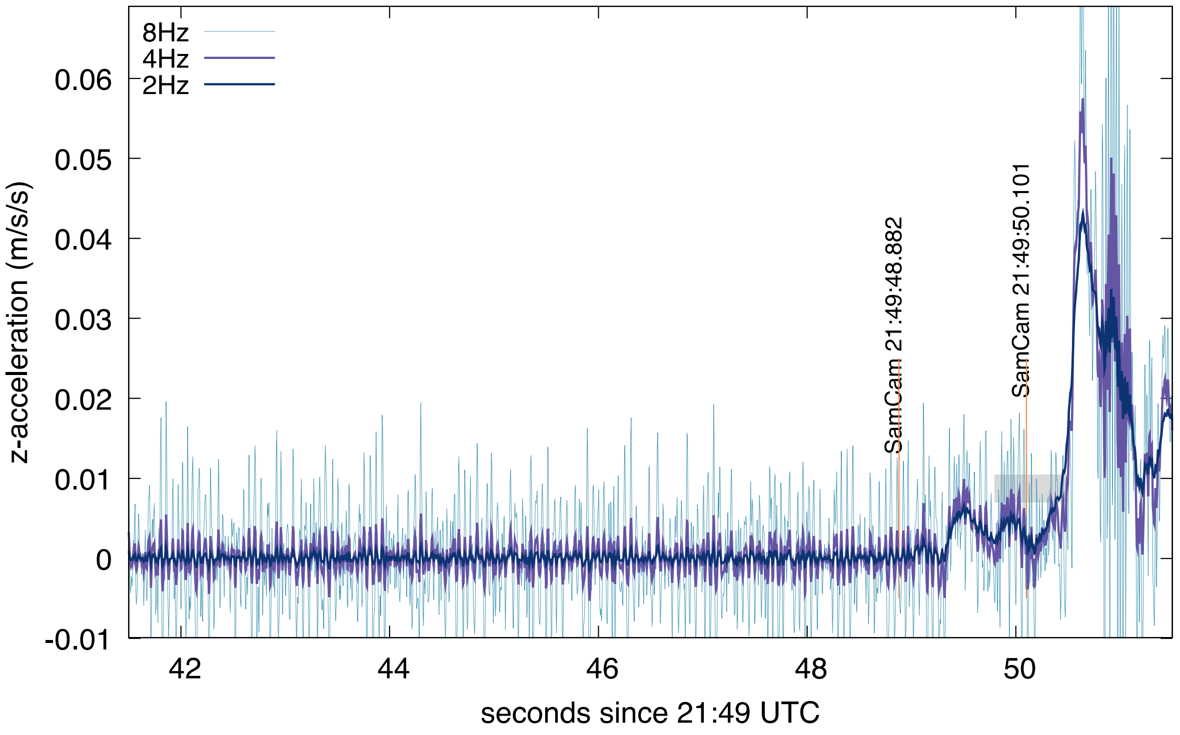

Fig S5. **The measured accelerations during contact and the derived depth profile**. Accelerations into the surface (*z* direction) measured by the spacecraft IMU at 200 Hz and analyzed with 2, 4, and 8 Hz lowband frequency cutoffs. Orange lines denote SamCam images taken before and after contact.

Table S1. Timeline of events during the sampling maneuver. See also Fig. 2 for a graphical representation. Times are in Universal Time Coordinated (UTC) at the spacecraft, and the significant digits indicates the precision to which the times are known. SamCam images are taken every ~1.2 s; none were taken during the 49th second of 21:49, and thus there is no SAM49.

UTC Time	Event	Description
21:49:48.882	SamCam image collected. (SAM48)	SamCam image before indication of surface interaction from IMU. Image id: 20201020T214948S882_sam
21:49:49.3±0.10	IMU change	First indication of surface interaction from IMU
21:49:49.795 to 21:49:49.895	TAGSAM flush with, and penetrating, the surface	Based on depth at SAM50 and integration of IMUs, this is the range of time for flush contact and start of penetration
21:49:49.680	NavCam 2 image	Last NavCam 2 image before gas release
21:49:50.101	SamCam image (SAM50)	SamCam image showing surface contact before gas release Image id: 20201020T214950S101_sam
21:49:50.421	Gas bottle open	Time of gas bottle opening command
21:49:50.5±0.10	IMU change	IMU begins registering force from gas release
21:49:50.66±0.10	Gas release detected	Time that measured pressure in gas bottles decreased.
21:50:50.680	NavCam 2 image	First NavCam 2 image after gas release

Table S2. Ellipse-fitting of TAGSAM before and after contact. Two images were selected for analysis of the TAGSAM orientation, with one before contact at 21:49:46.466 UTC and one after at 21:49:50.101 UTC (SAM50). Each was measured twice with the number in parentheses indicating the different instance. The ellipses' semi-major and semi-minor axes are listed in units of pixels. The azimuth indicates the orientation of the long-axis in degrees counter-clockwise from the +*y* axis of the image, and the tilt is the arccos of the ratio of the axes lengths.

Image time	Semi-major axis	Semi-minor axis	Azimuth	Tilt
21:49:46.466 (1)	147.5	146.0	26.9	8.3
21:49:46.466 (2)	148.05	146.1	26.3	9.1
21:49:50.101 (1)	145.7	140.1	90.7	15.9
21:49:50.101 (2)	146.0	140.9	89.4	15.1

Movie S1. Surface changes around the circumference of the TAGSAM head. Close-up view of the sample collection site just before contact at 21:49:48.882 UTC and at 21:49:50.101 UTC with a yellow boundary around the 0.51 m^2 area that envelopes surface disturbance.

Data S1.
The raw telemetry in the z direction for 21:49:49 to 21:49:50.5 UTC in units of seconds after 21:49 UTC Z acceleration in m/s/s.
